# Functional Interchangeability of Late Domains, Late Domain Cofactors and Ubiquitin in Viral Budding

**DOI:** 10.1371/journal.ppat.1001153

**Published:** 2010-10-21

**Authors:** Maria Zhadina, Paul D. Bieniasz

**Affiliations:** 1 Aaron Diamond AIDS Research Center and Laboratory of Retrovirology, the Rockefeller University, New York, New York, United States of America; 2 Howard Hughes Medical Institute, New York, New York, United States of America; Fred Hutchinson Cancer Research Center, United States of America

## Abstract

The membrane scission event that separates nascent enveloped virions from host cell membranes often requires the ESCRT pathway, which can be engaged through the action of peptide motifs, termed late (L-) domains, in viral proteins. Viral PTAP and YPDL-like L-domains bind directly to the ESCRT-I and ALIX components of the ESCRT pathway, while PPxY motifs bind Nedd4-like, HECT-domain containing, ubiquitin ligases (e.g. WWP1). It has been unclear precisely how ubiquitin ligase recruitment ultimately leads to particle release. Here, using a lysine-free viral Gag protein derived from the prototypic foamy virus (PFV), where attachment of ubiquitin to Gag can be controlled, we show that several different HECT domains can replace the WWP1 HECT domain in chimeric ubiquitin ligases and drive budding. Moreover, artificial recruitment of isolated HECT domains to Gag is sufficient to stimulate budding. Conversely, the HECT domain becomes dispensable if the other domains of WWP1 are directly fused to an ESCRT-1 protein. In each case where budding is driven by a HECT domain, its catalytic activity is essential, but Gag ubiquitination is dispensable, suggesting that ubiquitin ligation to trans-acting proteins drives budding. Paradoxically, however, we also demonstrate that direct fusion of a ubiquitin moiety to the C-terminus of PFV Gag can also promote budding, suggesting that ubiquitination of Gag can substitute for ubiquitination of trans-acting proteins. Depletion of Tsg101 and ALIX inhibits budding that is dependent on ubiquitin that is fused to Gag, or ligated to trans-acting proteins through the action of a PPxY motif. These studies underscore the flexibility in the ways that the ESCRT pathway can be engaged, and suggest a model in which the identity of the protein to which ubiquitin is attached is not critical for subsequent recruitment of ubiquitin-binding components of the ESCRT pathway and viral budding to proceed.

## Author Summary

The release of an enveloped virus particle from an infected cell requires the separation of the viral and cell membranes. Many enveloped viruses accomplish this by parasitizing a set of cellular proteins, termed the ESCRT pathway, that normally separates cellular membranes from each other. In some cases, viral structural proteins encode peptides motifs that bind directly to, and thereby recruit, the ESCRT machinery. Alternatively, viruses can recruit enzymes, termed ubiquitin ligases, that bind to other proteins, and catalyze the addition of ubiquitin to them. It has, heretofore, been somewhat unclear precisely how the recruitment of ubiquitin ligases leads to the engagement of the ESCRT machinery. We show that the simple recruitment of a fragment of a ubiquitin ligase that is responsible for the addition of ubiquitin to other proteins is sufficient to drive virus particle release, even when it is not possible to attach ubiquitin to viral proteins. Paradoxically, we also found that simple attachment of ubiquitin to the same viral protein can also drive particle release. These results show that there is flexibility in the ways in which the ESCRT machinery can be recruited and how ubiquitin can be co-opted to enable this.

## Introduction

The membrane scission event that separates the lipid membrane of nascent enveloped virions from host cell membranes is, in many cases, an orchestrated event requiring the participation of the class E vacuolar protein sorting (VPS), or endosomal sorting complex required for transport (ESCRT) pathway. Ordinarily, the ESCRT pathway induces topologically equivalent cellular membrane scission events including the biogenesis of multivesicular bodies (MVBs) [1], [2] and the membrane abscission event at the conclusion of cell division [3], [4]. Components of the pathway can be recruited, either directly or indirectly, through the action of short peptide motifs called late (L-) domains in viral structural proteins [5], [6]. Three classes of viral L-domains and cognate cofactors have been defined thus far: PT/SAP motifs bind Tsg101 [7], [8], [9], [10], LxxLF or YPXL motifs bind ALIX [11], [12], [13], and PPxY domains bind Nedd4-like HECT ubiquitin ligases [14], [15], [16], [17], [18]. Disruption of late domain function results in the failure of membrane scission and the accumulation of assembled virions that remain tethered to the surface of the host cell by a continuous membrane.

The ESCRT machinery is composed of ∼25 proteins, many of which participate in the formation of several multiprotein complexes, known as ESCRT-0, -I, -II, -III [19], [20], [21]. ESCRT-III components are thought to drive the membrane scission event [22], [23], [24], [25] and appear to be generally required for L-domain-dependent viral budding [7], [11], [12], [13], [26]. In contrast, other components of the ESCRT-pathway appear to be required in an L-domain specific way. For example, PTAP-dependent budding is especially sensitive to ESCRT-I perturbation, while YPXL-dependent budding is especially sensitive to ALIX depletion. Since ALIX interacts directly with ESCRT-III via its Bro1 domain [11], [12], [13], [27], [28] and ESCRT-I indirectly interacts with ESCRT-III via ALIX and/or ESCRT-II, [11], [12], [13] these observations suggest that YPXL and PTAP motifs access the same core scission machinery via alternative routes.

In contrast, it has remained somewhat unclear how PPxY motifs access the scission machinery. Overexpression of certain HECT ubiquitin ligases that bind directly to PPxY or other motifs can markedly stimulate budding, and the catalytic activity of the HECT domain is essential for this activity [17], [29], [30], [31]. Indeed, overexpression of catalytically inactive or truncation mutants of the HECT ligase WWP1 inhibits PPxY-dependent budding [17], [29]. Some components of the ESCRT pathway are also required for PPxY-induced budding [7], [31], [32]. However, the precise means by which HECT ligase recruitment subsequently results in the engagement of the ESCRT machinery is not completely defined. One model invokes direct ubiquitination of Gag as the key event. This notion derives from observations that several components of the ESCRT pathway are thought to recognize ubiquitinated cargo through various low affinity ubiquitin-binding domains [7], [33], [34], [35], [36], [37] and that monoubiquitination of cellular cargos can serve as a signal for endosomal trafficking and delivery to the lysosome [21], [38], [39]. Indeed, several observations are consistent with the notion that ubiquitination of retroviral Gag promotes virus particle release. For example, studies have noted an enrichment of free ubiquitin in retrovirus particles, and ubiquitinated Gag species have also been detected therein [14], [40], [41], [42], [43]. Additionally, late budding defects have been observed in cells treated with proteasome inhibitors, perhaps due to the depletion of free ubiquitin [14], [44], [45]. Mutation of multiple ubiquitin acceptor lysine residues in Gag has been shown to inhibit particle production by retroviruses [46], [47]. Finally, direct fusion of ubiquitin to the C-terminus of Gag proteins has been shown to alleviate inhibition of particle release imposed by proteasome inhibitors, or to obviate the requirement for an L-domain in particle release [44], [48].

Other observations suggest that PPxY and ubiquitin ligase-dependent budding may involve mechanisms other than direct Gag ubiquitination. In particular, overexpression of wild-type WWP1 stimulates PPxY-dependent particle production by a lysine-free Gag protein [29] in the absence of detectable Gag ubiquitination. This finding suggests the possibility that HECT ligases may promote budding by catalyzing the ubiquitination of specific trans-acting host factors, rather than Gag. Additionally, a HECT-truncated WWP1 protein, lacking the entire HECT domain, inhibits murine leukemia virus (MLV) budding more potently than the full length WWP1 protein with a disrupted active site [17], suggesting that HECT domains may possess activities other than ubiquitin conjugation that are important for their function in viral budding. Moreover, HECT domains localize to aberrant endosomal (so called class E) compartments induced by overexpression of catalytically inactive ATPase VPS4 [17], which is required for the disassembly of ESCRT complexes after each round of budding [49], [50]. Since many VPS factors are trapped on VPS4-induced compartments, HECT domains may be recruited to these compartments by interaction with VPS proteins, either directly or through unidentified bridging factors. It has also been reported that HECT ubiquitin ligases can bind to, and/or catalyze the ligation of ubiquitin to, certain class E VPS factors [31], [32], [51]. Thus, the ubiquitin ligases might act as recruitment factors rather than, or in addition to, conjugating ubiquitin to key target proteins.

In this study we investigated the role of PPxY motifs, HECT ubiquitin ligase domains and ubiquitin in viral budding, using a lysine-free viral protein from the prototypic foamy virus (PFV), in which the attachment of ubiquitin to Gag can be rather precisely controlled. We show that the catalytic activity of a variety of HECT domains, targeted to a PPxY motif in assembling particles via a common C2/WW domain fragment of WWP1, is essential for their ability to promote PPxY-dependent VLP release. In each case, however, Gag ubiquitination is dispensable for their activity. Rather, the ability of the chimeric ubiquitin ligases to promote budding correlated broadly, albeit imperfectly, with their ability to catalyze autoubiquitination, Moreover, we show that artificial recruitment of an isolated HECT domain can also stimulate budding, while a HECT domain becomes dispensable for PPxY motif dependent budding if the C2/WW domains of WWP1 are directly linked to the C-terminal domain of Tsg101, an ESCRT-I subunit. Finally, we demonstrate that direct fusion of a single ubiquitin moiety to the C-terminus of PFV Gag is also capable of promoting budding, in a manner that recapitulates the ESCRT protein requirement for budding induced by PPxY-dependent ubiquitin ligase recruitment in the absence of ubiquitin acceptors in Gag. These results support a model in which PPxY motif-induced HECT ubiquitin ligase recruitment leads to the deposition of ubiquitin at or near the site of viral budding. However, the identity of the protein to which ubiquitin is attached, be it Gag or a bystander protein, perhaps including the HECT ubiquitin ligase itself, does not appear to be critical in order for subsequent recruitment of ubiquitin-binding class E VPS proteins and viral budding to proceed.

## Results

### Chimeric ubiquitin ligases encoding a panel of HECT domains stimulate PPxY-dependent budding with variable efficiency

**Figure 1:**
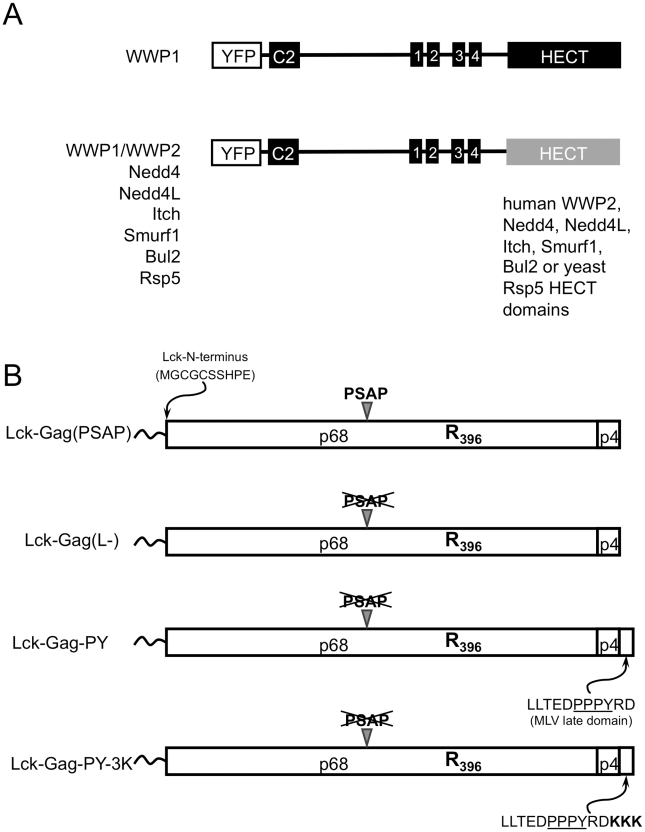
Constructs used in this study. (A) Schematic representation of YFP-fused chimeric ubiquitin ligases containing the membrane targeting (C2) and late domain binding (WW) domains of WWP1 and the indicated catalytic HECT domains. Wild type and catalytically inactive mutant versions of each HECT ligase were constructed. (B) Schematic representation of the PFV Gag-derived proteins used in this study, containing a single K396R mutation, an Lck-derived membrane targeting peptide appended to the N-terminus, and the indicated late domains.

To ascertain what properties of HECT domains are important for stimulation of virus particle release, we compared the properties of a panel of HECT domains. Nine members of the Nedd4-like HECT ubiquitin ligase family have been described in humans and these have the same domain organization as a single prototype member of this family in yeast, namely Rsp5 (reviewed in [52]). Specifically, an N-terminal C2 domain directs the protein to membranes, a central cluster of ‘WW” domains binds ligands, such as PPxY motifs, and a C-terminal HECT domain harbors the E3 ubiquitin ligase activity. Some of the intact ubiquitin ligases have been shown to vary in their ability to promote PPxY-dependent MLV virion release, due at least in part to differences in the affinities of their WW domains for the MLV L-domain [17], but whether the various the C-terminal HECT domains are equivalently able to induce particle release has not been investigated. We reasoned that variation in the ability of HECT domains to stimulate virus budding, correlated with a given property of the HECT domains, might suggest properties that are important for inducing virion release. Since WWP1 has been previously shown to be efficiently recruited by a number of PPxY-type L-domains, including that of MLV [17], we constructed a panel of chimeric ubiquitin ligases, consisting of membrane targeting and PPxY motif-binding domains (C2 and WW domains) of human WWP1, coupled to various catalytic HECT domains derived from human WWP2, Nedd4, Nedd4L, Itch, Smurf1, Bul2 or yeast Rsp5 HECT ligases (Fig. 1A).

To determine whether these chimeric ubiquitin ligases could support viral budding, we co-expressed each of them with a plasmid expressing a modified PFV Gag protein. Importantly, PFV Gag offers the advantage that it is naturally almost devoid of lysine resides. While PFV Gag normally requires a cognate Env protein for particle release, we have previously shown that appending a myristoylated, palmitoylated peptide from Lck at its N-terminus can overcomes this requirement by directing PFV Gag to the plasma membrane and thereby allowing the generation of extracellular particles in the absence of any other viral protein [29]. Throughout this study we used this N-terminally modified Gag protein, termed Lck-Gag, bearing a K396R mutation that renders the PFV Gag completely lysine-free. Examples of engineered variants of this Gag protein are illustrated in Fig. 1B, and include those that is otherwise unmodified and encode the natural PSAP late domain (Lck-Gag(PSAP)), a PSAP mutant that contains no known L-domain (Lck-Gag(L-)) or another variant that has a PPxY late domain derived from MLV Gag appended to its C-terminus (Lck-Gag-PY, Fig. 1B). In addition, we used an Lck-Gag-PY derivative containing three lysine residues adjacent to a PPxY late domain (Lck-Gag-PY-3K) to assess HECT ligase-induced Gag ubiquitination ([29], illustrated in Fig. 1B).

**Figure 2:**
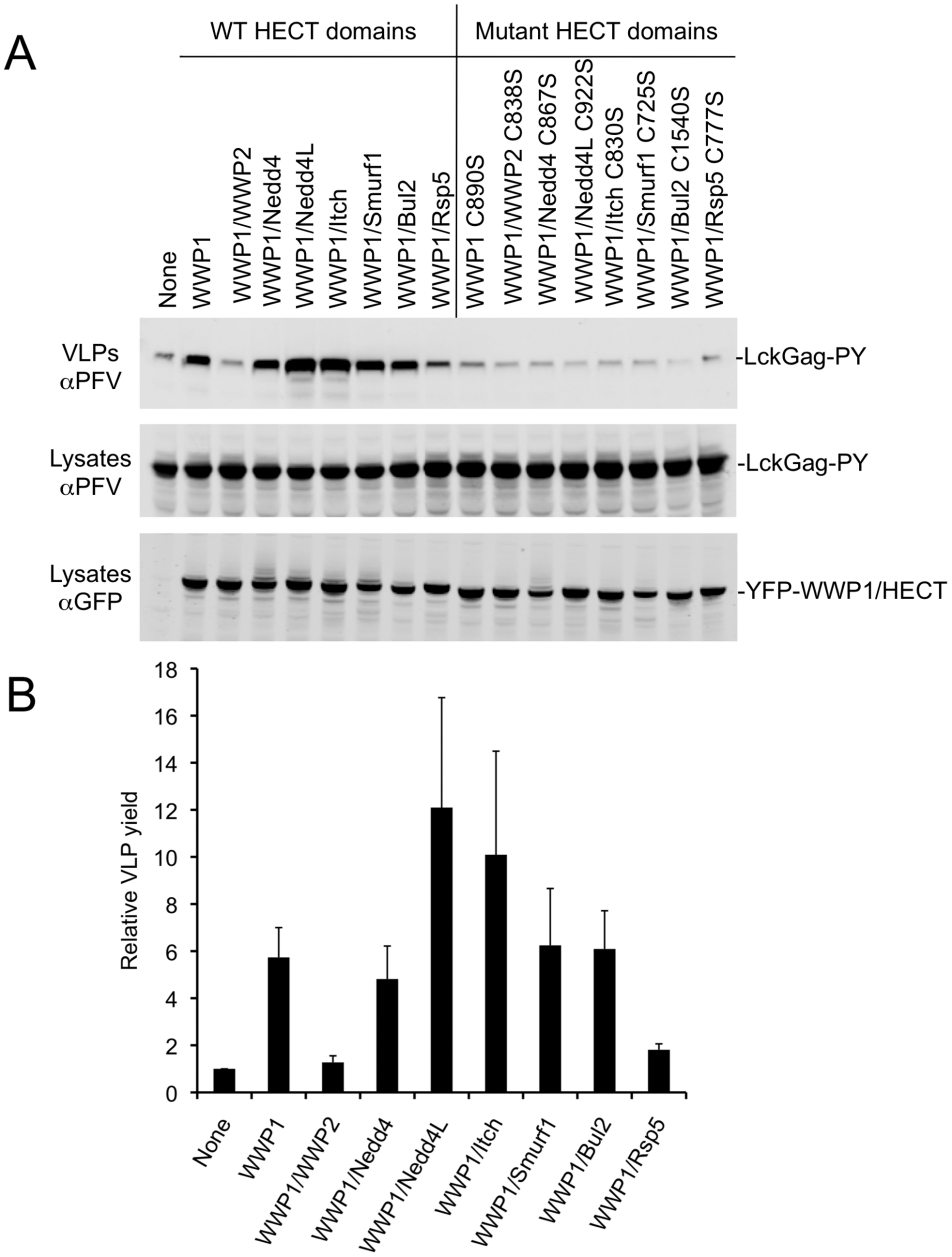
Stimulation of PPxY-dependent VLP production by chimeric HECT ubiquitin ligases. (A) Quantitative Western blot (LICOR) analysis of VLP release from 293T cells co-expressing Lck-Gag-PY and either YFP alone (None) or the indicated YFP-fused WWP1 C2/WW domains linked to the indicated HECT domains. Note that the unfused YFP is not visible in the “None” lane because it migrates to a different position on the blot. (B) Quantitation of Lck-Gag-PY protein in particles by quantitative Western blot analysis (LICOR). Values plotted are the levels of VLP associated Lck-Gag-PY protein generated in the presence of the indicated YFP-fused chimeric ubiquitin ligase, relative to that generated in the presence of YFP only (None). Data represent the mean and standard deviation of four independent experiments.

Overexpression of ubiquitin ligases encoding a variety of HECT domains (WWP1 itself, WWP1/Nedd4, WWP1/Nedd4L, WWP1/Itch, WWP1/Smurf1, or WWP1/Bul2) stimulated PPxY-dependent budding of lysine-free Lck-Gag-PY (Fig. 2A,B). Conversely, WWP1/WWP2 and WWP1/Rsp5 did not stimulate budding or had marginal activity. The strongest stimulation was observed using chimeric ligases containing the Nedd4L and Itch HECT domains. Importantly, overexpression of chimeric ligases in which the catalytic cysteine was mutated to serine, failed to stimulate PPxY-dependent particle release (Fig. 2A), indicating that the catalytic activity of each HECT domains was required, even when the viral structural proteins lack ubiquitin acceptors.

### Stimulation of Lck-Gag budding and HECT ubiquitin ligase autoubiquitination

**Figure 3:**
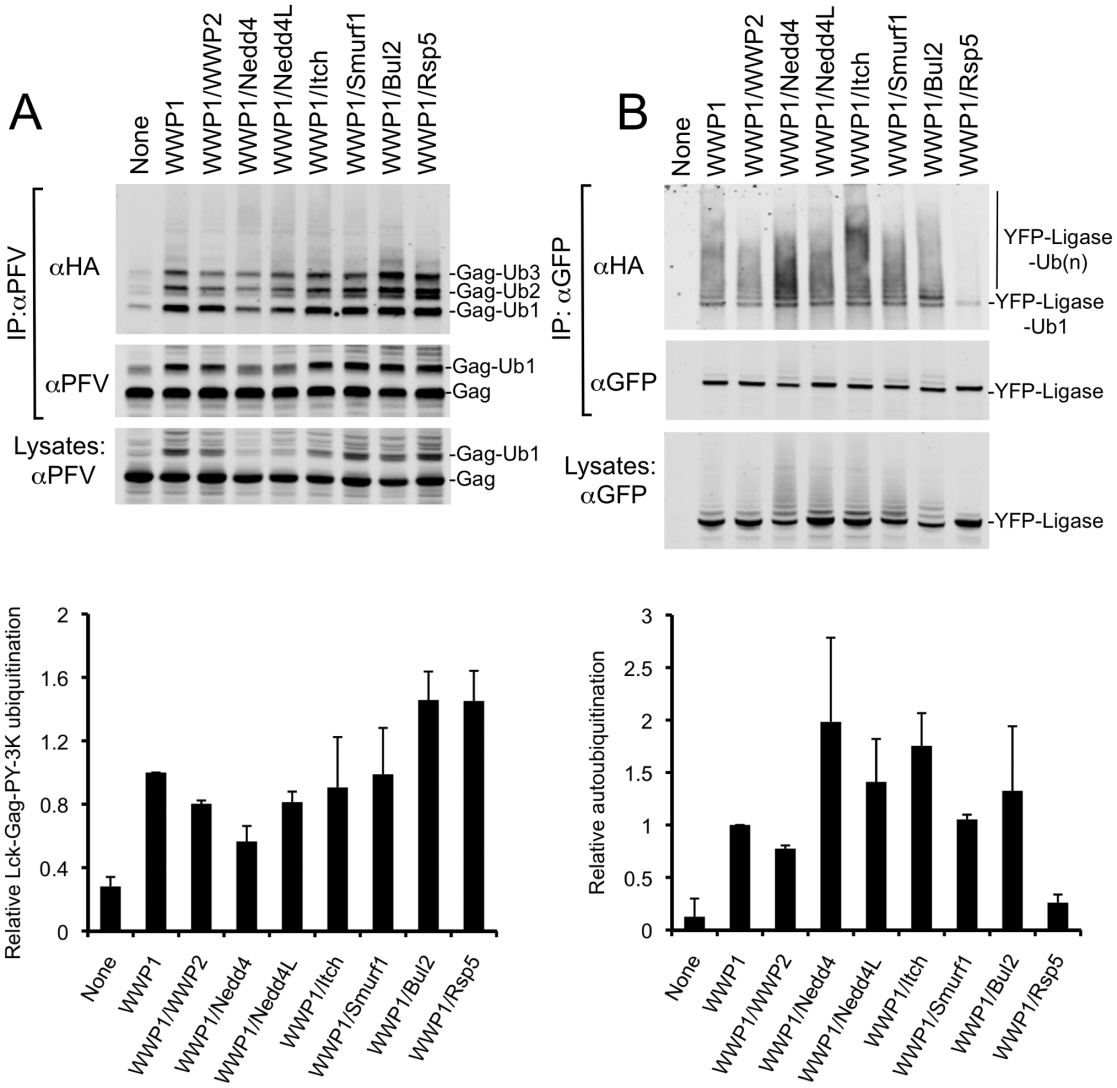
Chimeric HECT ligases catalyze varying levels of Lck-Gag-PY-3K ubiquitination and autoubiquitination. Quantitative Western blot analysis of lysine-containing Lck-Gag-PY-3K proteins (A) and chimeric HECT ligase proteins (B), immunoprecipitated from 293T cells following cotransfection with plasmids expressing Lck-Gag-PY-3K, HA-ubiquitin, and the indicated chimeric YFP-WWP1/HECT ubiquitin ligases. The αPFV Gag immunoprecipitates were probed with an αHA monoclonal antibody (A, top panel) or αPFV serum (A, middle panel). The αGFP immunoprecipitates were probed with αHA (B, top panel) and αGFP (B, middle panel) monoclonal antibodies. Alternatively, unfractionated cell lysates were probed with αPFV serum or αGFP antibody (A and B, bottom panels). Lanes marked “none” contain immunoprecipitates or lysates from cells transfected with unfused YFP in place of a YFP-fused ubiquitin ligase. Note that the unfused YFP is not visible in these lanes because is migrates to a different position on the blot. Charts below each plot show quantitation (mean+SD of two experiments) of the total signals in each lane of the blots in the upper panels that were probed for HA-ubiquitin. The levels of Gag ubiquitination and autoubiquitination are presented relative to that observed in the presence of intact WWP1, which was assigned a value of 1.

To assess the relative catalytic activities of the chimeric HECT ligases, and assess whether this correlated with their differential ability to promote budding, we compared their abilities to carry out autoubiquitination and to ubiquitinate a Gag substrate encoding three lysine residues in close proximity to a PPxY late domain (Lck-Gag-PY-3K, see Fig. 1B). To accomplish this, we immunoprecipitated either Gag or HECT ubiquitin ligases from 293T cell lysates, prepared 36 hours after co-transfection with plasmids expressing Lck-Gag-PY-3K, HA-tagged ubiquitin, and each of the YFP-fused chimeric HECT ligases. Cell lysates were prepared using denaturing, detergent-rich buffer (containing 0.5% SDS) to ensure dissolution of protein complexes, and ubiquitinated species were detected by immunoprecipitation with either αPFV Gag or αGFP antibodies followed by immunoblot analysis of the precipitates with an αHA antibody (Fig. 3).

Each of the chimeric HECT ubiquitin ligases was able to reasonably efficiently catalyze the addition of 1 to 3 ubiquitin moieties to the Lck-Gag-PY-3K substrate (Fig. 3A, upper panels). There was some variation in the ability of the HECT domains to catalyze the ligation of ubiquitin to Lck-Gag-PY-3K, with WWP1/Rsp5 and WWP1/Bul2 catalyzing the highest and WWP1/Nedd4 the lowest levels of ubiquitin ligation to Lck-Gag-PY-3K (Fig. 3A). However, there was no correlation between the extent to which each HECT domain stimulated Lck-Gag-PY-3K ubiquitination (Fig. 3A) and the degree to which it stimulated the release of VLPs assembled using Lck-Gag-PY or Lck-Gag-PY-3K (Fig. 2A and data not shown). For example, WWP1/Bul2 and WWP1/Nedd4, which induced the highest and lowest levels of Gag ubiquitination, respectively (Fig. 3A), stimulated budding to a similar extent (about 6-fold, Fig. 2A). Moreover, WWP1/Rsp5, which efficiently catalyzed Gag ubiquitination (Fig. 3A), enhanced particle release only marginally (Fig. 2A), much less efficiently than the WWP1/Nedd4L that induced comparatively modest levels of Gag ubiquitination (Fig. 3A).

We observed a better, albeit imperfect, correlation between the ability of the chimeric HECT ligases to catalyze autoubiquitination and to stimulate VLP production (Fig. 3B, Fig. 2B). Chimeric ligases that strongly promoted Lck-Gag-PY VLP release (e.g. WWP1/Itch and WWP1/Nedd4L) were more heavily autoubiquitinated, while those that failed or only marginally promoted VLP release (WWP1/WWP2 and WWP1/Rsp5, Fig. 2) exhibited the lowest levels of autoubiquitination (Fig. 3B). The correlation was imperfect, however, since WWP1/Nedd4, which moderately enhanced particle release (Fig. 2), was consistently highly auto-ubiquitinated (Fig. 3B). Notably, there was no correlation between the ability of the HECT ubiquitin ligases to catalyze autoubiquitination, and their ability to catalyze ubiquitin ligation to Lck-Gag-PY-3K (Fig. 3A, B). Overall, these data confirm our previous finding that direct Gag ubiquitination is dispensable for HECT ligase-dependent budding [29] and further indicates that intrinsic catalytic activity of the HECT ubiquitin ligases is critical for their ability to stimulate budding.

### Reciprocal exchange of HECT ubiquitin ligase and Tsg101 domains results in functional hybrid L-domain cofactors that can promote viral budding

**Figure 4:**
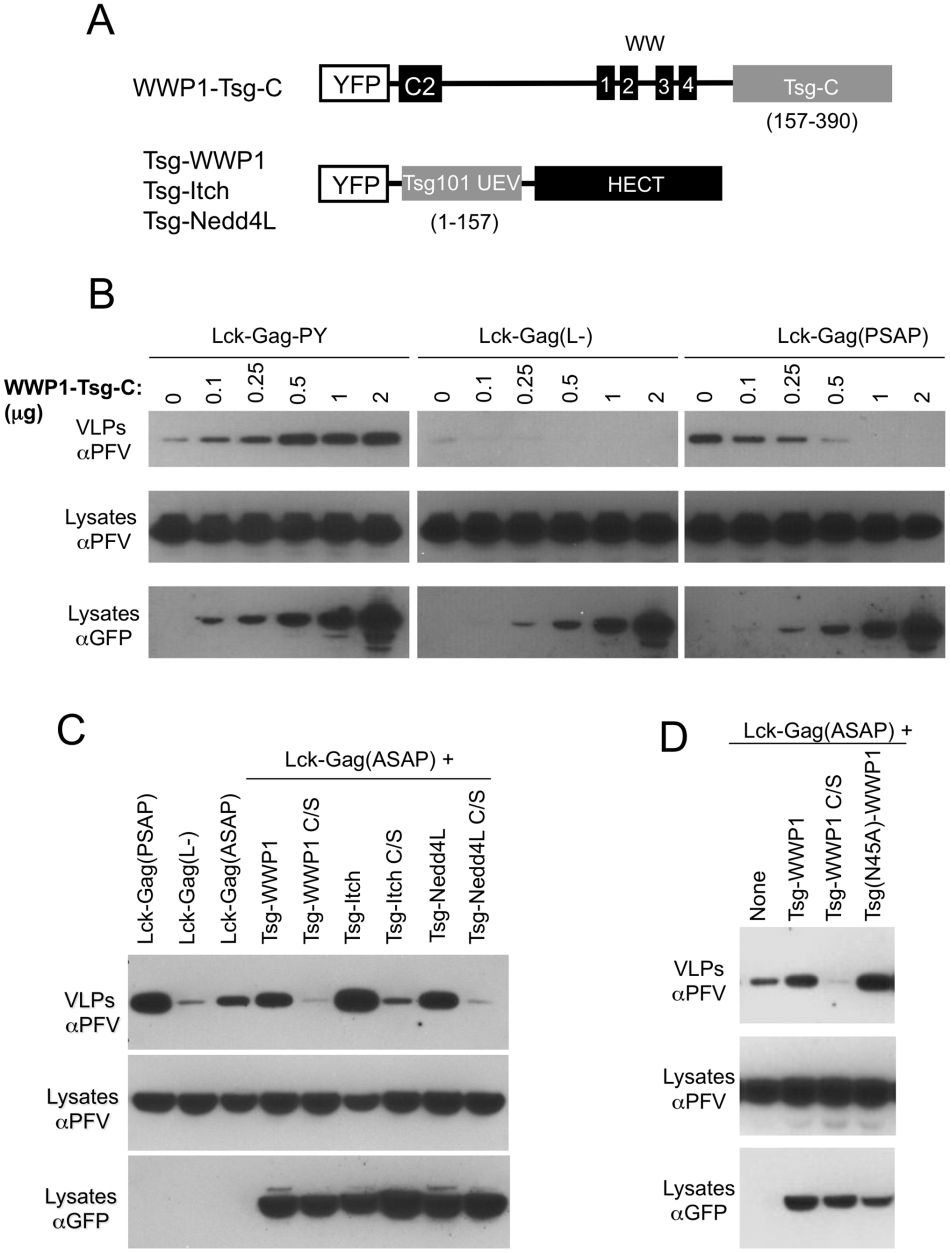
Exchange of functional domains in L-domain cofactors. (A) Schematic representation of chimeric proteins designed to recruit ESCRT-I to PPxY motif (WWP1-Tsg-C), and isolated catalytic domain of a HECT ubiquitin ligase to a PTAP motif (Tsg-WWP1, Tsg-Itch and Tsg-Nedd4L). (B) Stimulation of PPxY-dependent budding in the absence of a HECT domain by direct recruitment of ESCRT-I. Specifically, VLP production from 293T cells expressing Lck-Gag-PY, Lck-Gag(L-) or Lck-Gag(PSAP) and increasing amounts of YFP-fused WWP1-Tsg-C (the amount of cotransfected WWP1-Tsg-C expression plasmid (in µg) is indicated) was assessed by western blotting. (C,D) VLP production from 293T cells expressing Lck-Gag containing either a wild-type (Lck-Gag(PSAP)), inactive Lck-Gag(L-), or attenuated Lck-Gag(ASAP) late domain and the indicated WT or catalytically inactive mutant (C/S) chimeric Tsg-WWP1, Tsg-Itch and Tsg-Nedd4L proteins was assessed by western blotting. The three leftmost lanes in (C) and the single leftmost lane in (D) contain lysates from cells transfected with unfused YFP as a control in place of a YFP-fused Tsg-HECT protein. Note that the unfused YFP is not visible in these lanes because it migrates to a different position on the blot.

We next asked whether the need to recruit a HECT domain in the context of PPxY/WWP1 interaction was necessary for particle release, or whether the HECT domain could be bypassed by direct recruitment of putative downstream effectors. Additionally, we asked whether recruitment of a HECT domain in the absence of the other domains (C2 and WW) found in the Nedd4-like family of proteins was sufficient to stimulate particle budding. To accomplish this, we constructed hybrid L-domain cofactors in which the essential domains were split and linked to putatively complementing domains in another L-domain cofactor (Fig. 4A). Specifically, Tsg101 is a core component of ESCRT-I and contains two domains that are functionally important with respect to viral budding. The N-terminal ubiquitin E2 variant (UEV) domain interacts directly with P(T/S)AP peptide motifs and ubiquitin [7], while the C-terminal portion of the protein is a key structural component of ESCRT-I, interacting with other components, e.g. VPS28 and VPS37 [53], [54], [55] and is essential to support Tsg101 dependent budding. We constructed an artificial putative chimeric L-domain cofactor in which the C2/WW domains of WWP1 were linked to the C-terminal portion of Tsg101 (Tsg-C) that constitutes the core structural component of ESCRT-I (residues 157–390, Fig. 4A). Notably, overexpression of this chimeric protein, termed WWP1-Tsg-C, stimulated Lck-Gag-PY particle release in a dose-dependent manner but had no effect on particle production by the L-domain-deficient Lck-Gag(L-) protein (Fig. 4B, left and middle panels). This chimeric protein, therefore, appeared capable of recruiting a functional ESCRT-I complex to PPxY L-domains and thereby stimulating particle production. Conversely, WWP1-Tsg-C overexpression inhibited Lck-Gag(PSAP) budding in a dose-dependent manner (Fig. 4B, right panel). We surmise that since this chimeric protein lacks the domains required for interaction with PT/SAP motifs, it acts as an inhibitor of PSAP-dependent budding by sequestering endogenous components (e.g. VPS28 and VPS37) into retargeted ESCRT-I complexes that can be recruited to PPxY, but not PT/SAP, L-domains. Thus, these experiments demonstrate that the requirement for a HECT domain (and, by inference, the requirement for ubiquitin ligation) in PPxY/ubiquitin ligase dependent viral budding can be bypassed, if an alternative link to the ESCRT machinery is provided.

In a reciprocal experiment, we asked whether the PPxY motif and the C2/WW domains of WWP1 could be functionally replaced in the context of HECT domain/ubiquitin dependent budding. In other words, we determined whether recruitment of a HECT domain is sufficient to stimulate particle release, in the absence of the other protein domains (C2 and WW) to which it would ordinarily be linked. Specifically, we attempted to redirect P(T/S)AP-dependent particle production through a HECT domain-dependent pathway, by constructing chimeric proteins, termed Tsg-WWP1, Tsg-Itch and Tsg-Nedd4L, that contained the N-terminal UEV domain (residues 1–157) of Tsg101 linked to one of the three respective HECT domains (Fig. 4A). To test the function of these artificial putative L-domain cofactors, we also constructed an attenuated “leaky” mutant of the PT/SAP motif in the Lck-Gag(PSAP) protein, namely Lck-Gag(ASAP), by mutating the first proline residue of the PSAP motif to alanine. In the context of the HIV-1 PTAP motif, such a mutation reduces the affinity for, but does not eliminate binding to the Tsg101 UEV domain [7]. Correspondingly, the budding of Lck-Gag(ASAP), was attenuated as compared to Lck-Gag(PSAP), but the ASAP motif clearly retained some weak residual ability to stimulate budding (Fig. 4C, leftmost three lanes), suggesting that it retains some residual ability to recruit the Tsg101 UEV domain.

Overexpression of Tsg-WWP1, Tsg-Itch or Tsg-Nedd4L, respectively) resulted in clear stimulation of Lck-Gag(ASAP) budding (Fig. 4C). Tsg101-Itch was the most potent of the three Tsg101-HECT proteins tested by this approach, and its overexpression resulted in a particle yield that matched or even exceeded that observed in the presence of the intact PSAP motif (Fig. 4C). In contrast, expression of catalytically inactive versions of Tsg-WWP1, Tsg-Itch or Tsg-Nedd4L inhibited rather than enhanced Lck-Gag(ASAP) particle production (Fig. 4C). Because the Tsg101 UEV domain contains ubiquitin-binding activity that might complicate the interpretation of these results, we repeated these experiments using a mutant Tsg101 UEV domain (N45A) that is defective for ubiquitin binding, linked to a WWP1 HECT domain. The mutant Tsg(N45A)-WWP1 fusion stimulated budding at least as efficiently as did the unmanipulated Tsg-WWP1 protein (Fig. 4D).

Overall, the experiments in Fig. 4 demonstrate that the domains of the PTAP and PPxY binding cofactors can be functionally split into modular, interchangeable domains that are (i) necessary for binding to the L-domain and (ii) interface with downstream effectors that are critical for budding. Most notably, these findings suggest that simple recruitment of a HECT domain to sites of particle budding, irrespective of its mode of recruitment, and in the absence of ubiquitin acceptors on the viral protein, is sufficient to stimulate particle release and that other HECT ubiquitin ligase domains are dispensable for budding.

### Direct fusion of ubiquitin to Lck-Gag promotes particle release

**Figure 5:**
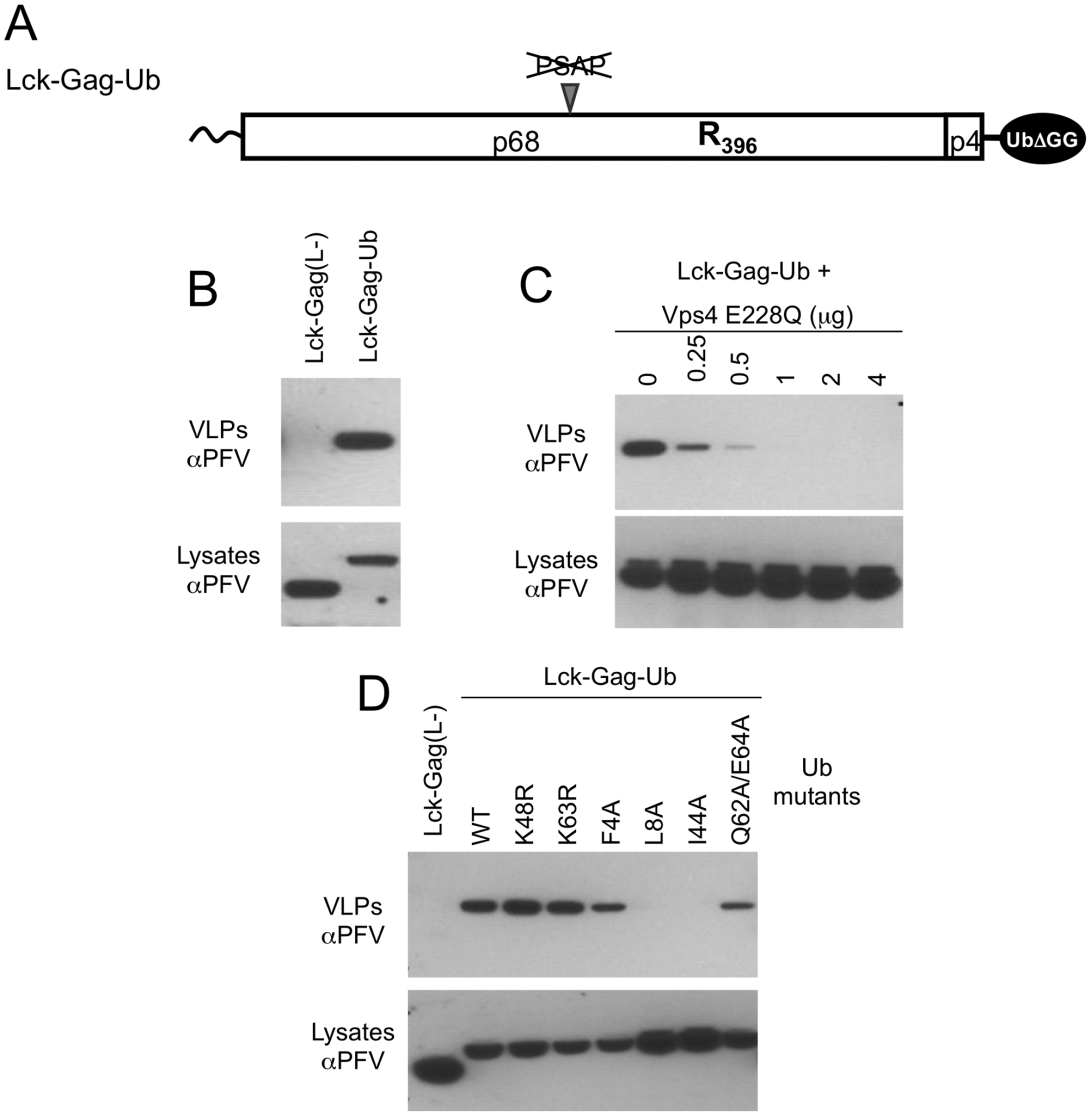
Direct fusion of ubiquitin to Lck-Gag stimulates L-domain-dependent and -independent budding. (A) Schematic representation of the Lck-Gag-Ub protein with a single ubiquitin moiety fused at the carboxyl terminus. Two glycine residues were removed from the C-terminus of ubiquitin to prevent its conjugation to other proteins. (B) VLP production from 293T cells expressing the Lck-Gag-Ub protein, or a control Lck-Gag(L-) protein was assessed by western blotting. (C) VLP production from 293T cells expressing Lck-Gag-Ub and increasing amounts of GFP-Vps4 (E228Q). (D) VLP release from 293T cells expressing Lck-Gag-Ub containing the indicated mutations in C-terminally fused ubiquitin.

The aforementioned experiments demonstrated that the requirement for a catalytically active HECT domain could be obviated by direct recruitment of ESCRT-I to a viral protein (Lck-Gag-PY) whose budding would normally be dependent on such recruitment. We next asked whether the requirement for HECT domain recruitment could similarly be obviated, in the context of a nearly identical viral protein, by simply depositing ubiquitin at the site of particle assembly, in the absence of ubiquitin ligase recruitment. To mimic the deposition of ubiquitin at sites of virion assembly, in the absence of ubiquitin ligase recruitment, we expressed an Lck-Gag protein, lacking L-domains, with a single ubiquitin appended at its C-terminus (Lck-Gag-Ub, Fig. 5A). Ubiquitin is normally conjugated to proteins by an isopeptide bond between the C-terminal glycine residue of ubiquitin and the ε-amino group of a lysine residue within the substrate protein. Therefore, to avoid aberrant conjugation of our Gag-ubiquitin chimeras to other proteins we deleted two glycine residues from the C-terminus of ubiquitin (Fig. 5A).

Cells expressing ubiquitin-fused, but L-domain-deficient Gag (Lck-Gag-Ub) generated extracellular particles while those expressing the unfused, L-domain deficient counterpart Lck-Gag(L-) protein did not (Fig. 5B). Directly fused ubiquitin-dependent particle release was strongly inhibited, in a dose dependent manner, by expression of a catalytically inactive version of the ATPase VPS4 (Fig. 5C), indicating that the ESCRT pathway was required for Lck-Gag-Ub particle release. Thus, in the context of Lck-Gag, direct ubiquitin fusion appeared capable of substituting for a PSAP or PPxY containing L-domain. These results are similar to findings made by Joshi et al. who showed that direct fusion of ubiquitin to EIAV Gag can functionally substitute for the ALIX-binding YPDL L-domain encoded therein [48]. Similarly, we also found that ubiquitin-dependent budding was dependent on the ubiquitin hydrophobic patch residues (L8 and I44) and additionally, marginally dependent on residues (Q62 and E64) that have been implicated in ubiquitin-Tsg101 UEV domain interaction (Fig. 5D). However, lysine residues (K48 and K63) that are often important for the conjugation of further ubiquitin molecules could be mutated without affecting fused ubiquitin-dependent particle release (Fig. 5D).

### Ubiquitin and PSAP motifs synergize to stimulate Lck-Gag VLP release

**Figure 6:**
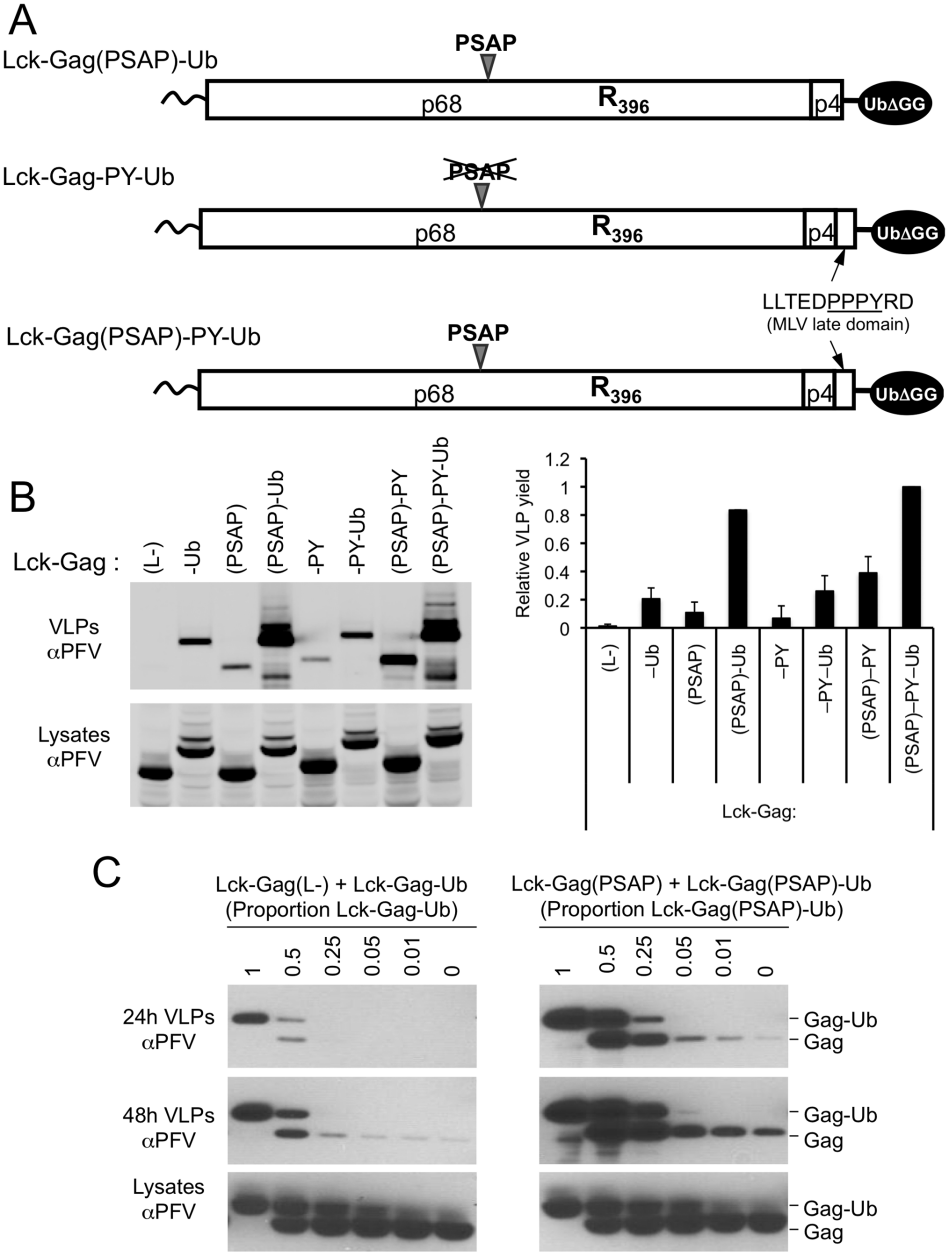
Synergy between a PSAP motif and ubiquitin during viral budding and effect of Gag-ubiquitin levels on particle release. (A) Schematic representation of some examples of the Lck-Gag proteins used in these experiments, encoding PSAP and/or PPxY L-domains and a single ubiquitin moiety fused at the carboxyl-terminus. (B) VLP release from by 293T cells expressing Lck-Gag proteins containing the indicated L-domains that were present alone or in combination with each other and/or directly fused ubiquitin (see panel A), measured by quantitative western blotting. The chart to the right of the blot shows the yield of VLPs (mean+SD of 2 experiments) and values are presented relative to the VLP yield obtained in the presence of both L-domains and ubiquitin (Lck-Gag(PSAP)-PY-Ub), which was assigned a value of 1. (C) VLP production from 293T cells expressing Lck-Gag(L-) or Lck-Gag(PSAP) proteins, where the total amount of Gag protein expressed was constant, but varying proportions carried a fused ubiquitin. Cells were transfected with equal total amounts of Lck-Gag(L-)+Lck-Gag-Ub (left panels) or Lck-Gag(PSAP)+Lck-Gag(PSAP)-Ub expression plasmids, and the indicated fraction of the total transfected plasmid mixture expressed the ubiquitin fused form of the Gag protein. VLPs were harvested both at 24h and 48h after transfection and subjected to western blot analysis with αPFV antiserum.

Next we analyzed the effect of combining L-domains and ubiquitin on VLP release. To accomplish this, Lck-Gag proteins containing various combinations of the L-domains and C-terminally fused ubiquitin (Fig. 6A) were expressed. Quantitative analyses revealed that directly fused ubiquitin-dependent (Lck-Gag-Ub) particle release was at least as efficient as that driven by PSAP (Lck-Gag(PSAP)) or PPxY (Lck-Gag-PY) L-domains (Fig. 6B). Moreover, and in contrast to the previous report with EIAV Gag [48], we found that the combined presence of fused ubiquitin and a PSAP L-domain (in Lck-Gag(PSAP)-Ub) resulted in strongly synergistic effects on particle release (Fig. 6B). Specifically, Lck-Gag(PSAP)-Ub generated ∼20-fold and ∼6-fold more particles than Lck-Gag(PSAP) and Lck-Gag-Ub, respectively (Fig. 6B). No such synergy was observed when a PPxY L-domain and ubiquitin were combined in the same Gag protein. In fact, the Lck-Gag-Ub and the Lck-Gag-PY-Ub generated extracellular particles with approximately the same efficiency (Fig. 6B). Less dramatic, but nonetheless synergistic enhancement of particle release was evident when PPxY and PSAP motifs were both present (in the absence of ubiquitin fusion, Fig. 6B). In this case, the presence of the PPxY motif (in Lck-Gag(PSAP)-PY) enhanced particle release approximately ∼5-fold as compared to the situation where the PSAP motif was the only L-domain (in Lck-Gag(PSAP), Fig. 6B). Overall these results are consistent with the notion that ubiquitin behaves essentially like an L-domain, and further suggests that it functions synergistically with a PT/SAP motif, and redundantly with a PPxY motif.

We next attempted to mimic a situation that is somewhat typical of retroviruses, where only a fraction of Gag expressed in cells carries ubiquitin. This was done by co-expressing ubiquitin-fused and unfused Lck-Gag proteins in varying proportions. When this was done in the context of a Lck-Gag proteins lacking a PSAP motif (by co-expressing Lck-Gag(L-) and Lck-Gag-Ub), particle production was most efficient when a large fraction of the total Lck-Gag protein carried ubiquitin, and no stimulation of particle production was detectable when less than 25% of the Gag protein carried fused ubiquitin (Fig. 6C, left panel). When similar experiments were done in the presence of a PSAP late domain, by co-expressing Lck-Gag(PSAP) and Lck-Gag(PSAP)-Ub, stimulation of particle release was observed when smaller fractions of Gag, as little as a few percent, carried ubiquitin (Fig. 6C, right panel). Nonetheless, larger fractions of ubiquitin fused Gag had larger stimulating effects on particle release. Thus, these experiments suggest that the greater the number of ubiquitin molecules that are present at sites of particle assembly, the more efficient is particle release; however, relatively modest amounts of ubiquitin can significantly enhance particle budding in the presence of a PSAP motif.

### PPxY L-domain-dependent and directly fused ubiquitin-dependent particle release are similarly dependent on particular ubiquitin binding class E VPS factors

Several class E vacuolar protein-sorting factors have been reported to possess ubiquitin binding activity (Table 1). Although the affinity of such domains for monoubiquitin is generally quite weak (K_d_>100µM), several class E factors form multiprotein complexes with several ubiquitin-binding surfaces, which could provide sufficient avidity for their retention at sites of virion assembly. Under such a scenario, efficient recruitment of ESCRT complexes might require deposition of relatively large numbers of ubiquitin molecules in the vicinity of the assembling particle, a notion that is consistent with the finding that a large fraction of Gag must carry ubiquitin to compensate for the absence of a late domain (Fig. 6C).

**Table 1:** Class E VPS factors and associated proteins encoding ubiquitin binding domains.

Protein	Domain type	Kd	Methods	References
Tsg101	UEV	∼500µM	SPR, NMR, structure	[7], [34], [59], [60]
Hrs	DUIM, VHS	∼300µM, ∼1.4mM	IP, SPR, structure	[34], [35], [36], [61], [62], [63]
STAM	UIM, VHS,	∼430µM, ∼220µM	IP, NMR, Y2H	[33], [64], [65], [66], this study
Eap45	GLUE	∼300µM	IP, SPR, structure	[37], [67], [68]
Eps15	UIM	∼0.36µM	IP, SPR	[35], [36]
ALIX	Unknown		IP, Y2H	[48], this study
CIN85	SH3		IP	[69]

SPR: surface plasmon resonance; NMR: nuclear magnetic resonance; IP: immunoprecipitation or bead based ‘pull-down’ assays; Y2H: yeast 2-hybrid.

**Figure 7:**
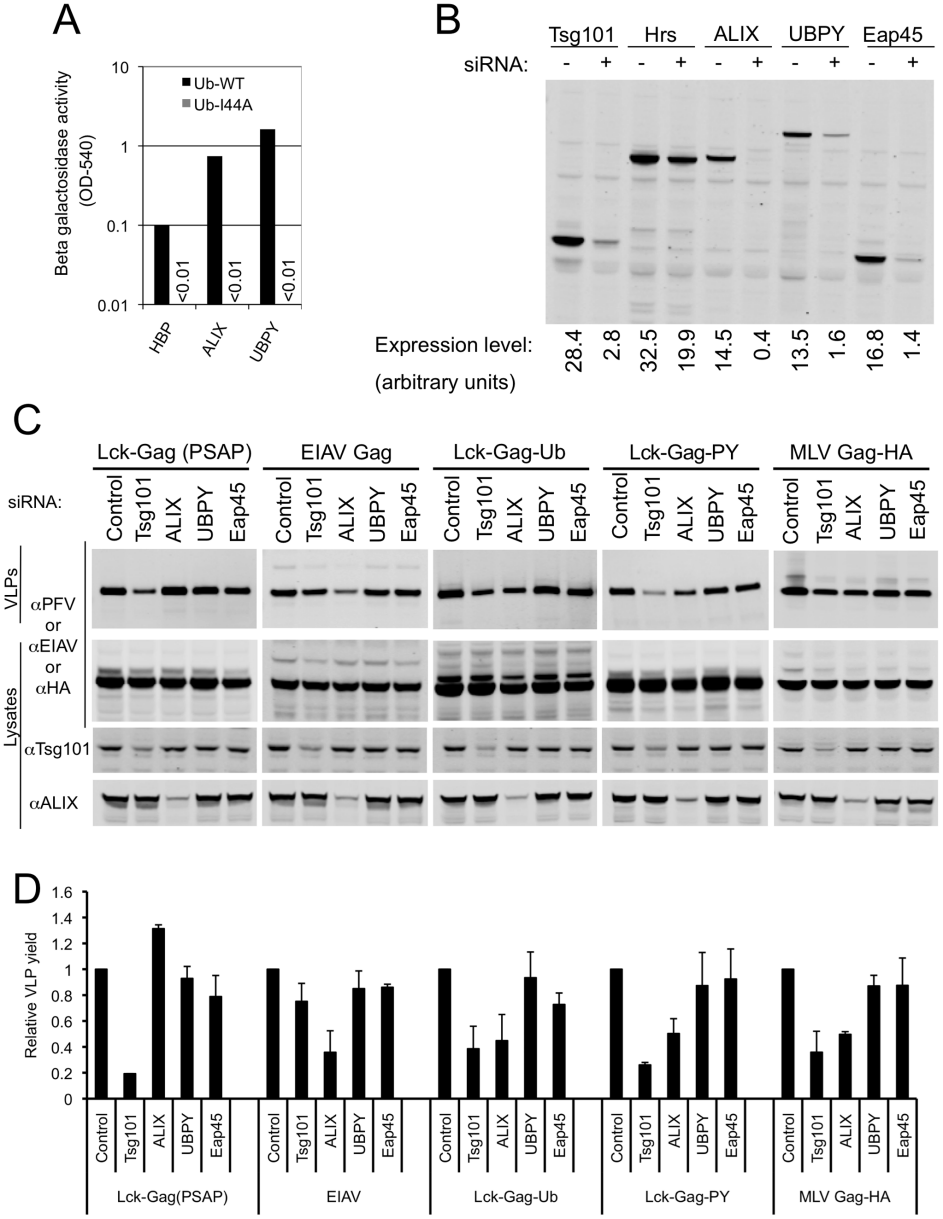
Role of ubiquitin-binding class E VPS factors in ubiquitin-dependent and PPxY-dependent particle release. (A) Yeast two-hybrid analysis of the interaction between the indicated class E VPS factors and ubiquitin containing either an intact (Ub WT) or disrupted (Ub I44A) hydrophobic patch. β-galactosidase expression was measured (as optical density at 540nm (OD540)) in Y190 cells transformed with the indicated Gal4-DNA binding domain-ubiquitin and VP16 activation domain (-HBP, -ALIX, and -UBPY) fusion constructs. Absence of a bar indicates background levels of β-galactosidase expression. A single representative of two independent experiments is shown. (B) Validation of siRNAs targeting ubiquitin-binding ESCRT-complexes or other class E VPS factors. Lysates of 293T cells transfected with GFP-Tsg101 or YFP-Hrs, -ALIX, -UBPY, or -Eap45 expression plasmids and siRNAs targeting either luciferase (−) or the specified class E VPS factors (+) were probed with an αGFP monoclonal antibody. (C) L-domain-specific inhibition of Gag budding by class E factor depletion. Quantitative Western blot analysis of VLP production from 293T cells transfected with plasmids expressing Lck-Gag(PSAP), EIAV Gag, Lck-Gag-Ub, Lck-Gag-PY or MLV Gag-HA and siRNAs directed against the indicated class E VPS protein. Corresponding cell lysates were also probed with antibodies to PFV, EIAV, HA, Tsg101 and/or ALIX, as appropriate. (D) Quantitation of VLP release following knockdown of the indicated class E VPS proteins. Values are plotted the mean+SD of two or three independent experiments and represent the levels of particles released relative to those released from cells transfected with control luciferase siRNAs, which was assigned a value of 1.

To determine which of the mammalian ESCRT complexes and associated proteins might be most important for ubiquitin dependent budding, we performed a directed yeast two-hybrid screen in which ubiquitin binding to a range of human class E VPS factors and associated proteins was surveyed. These included components of ESCRT-0 (Hrs, HBP/STAM), ESCRT-I (Tsg101, VPS28, VPS37A,B,C, Mvb12), ESCRT-II (Eap20, Eap30, Eap45) ESCRT-III (CHMP1A, 1B, 2A, 2B, 3, 4A, 4B, 4C, 5, 6), as well as several ESCRT-associated proteins or proteins that are known to bind to components of the class E VPS pathway (ALIX, LIP5, VPS4, UBPY,CMS, CIN85). Most of these proteins, including known ubiquitin binding factors (Table 1), gave either weak or non-specific signals. Since we were testing ubiquitin binding by each protein individually and outside of its natural context and in the absence of ESCRT complex partners, it was perhaps to be expected that this assay would fail to detect ubiquitin interactions in at least some instances. Nonetheless, HBP/STAM, ALIX, and UBPY binding gave robust signals in WT ubiquitin binding assays, and binding was abolished when the ubiquitin hydrophobic patch was mutated (I44A), (Fig. 7A).

We next determined the effect of siRNA mediated disruption of known ubiquitin-binding complexes, as well the additional ESCRT-associated factors that were positive in our yeast 2-hybrid survey (ALIX and UBPY), on PPxY-dependent and fused ubiquitin-dependent Lck-Gag budding. The core components of the known ubiquitin binding ESCRT complexes (ESCRT-0, ESCRT-I and ESCRT-II) were targeted using pools of four siRNAs directed to Hrs, Tsg101 and Eap45, respectively. The potency of the siRNA pools was estimated by cotransfecting them with plasmids expressing YFP-tagged target proteins, followed by quantitative western blotting. By these criteria the Tsg101, Eap45, ALIX and UBPY siRNAs appeared effective (Fig. 7B). However, knockdown of Hrs was inefficient, so its effect on budding could not be reliably assessed. Because antibodies to Tsg101 and ALIX were available, the level of endogenous proteins could also be monitored in these siRNA experiments. Quantitative western blotting analyses (examples are shown in Fig. 7C) indicated that Tsg101 and ALIX proteins were reduced to 38±9% and 16±4% of endogenous levels, respectively. Notably, control experiments showed that Lck-Gag(PSAP) particle release was specifically inhibited (∼5-fold) by Tsg101 siRNA, but only marginally affected by EAP45, ALIX and UBPY depletion (Fig. 7C,D), while EIAV Gag particle release was specifically inhibited (∼3-fold) by ALIX depletion, but not by depletion of the other ESCRT-associated proteins (Fig. 7C,D).

Ubiquitin-dependent (Lck-Gag-Ub) budding was modestly inhibited (∼3-fold) by depletion of either Tsg101 or ALIX but was barely affected by UBPY or Eap45 siRNAs (Fig. 7C,D), suggesting that ubiquitin binding to ESCRT-I and ALIX contributes to its ability to mediate particle release. This finding mirrors a previous report using ubiquitin fused to EIAV Gag [48]. Additionally, however, we further found that Lck-Gag-PY exhibited a similar pattern sensitivity to class E factor-targeting siRNAs, in that it was modestly sensitive to Tsg101 and ALIX but not Eap45 or UBPY siRNAs (Fig. 7C,D). Similarly, the budding of an MLV Gag protein, that carries the same PPxY L-domain was also modestly sensitive to depletion of Tsg101 and ALIX Fig. 7C,D).

**Figure 8:**
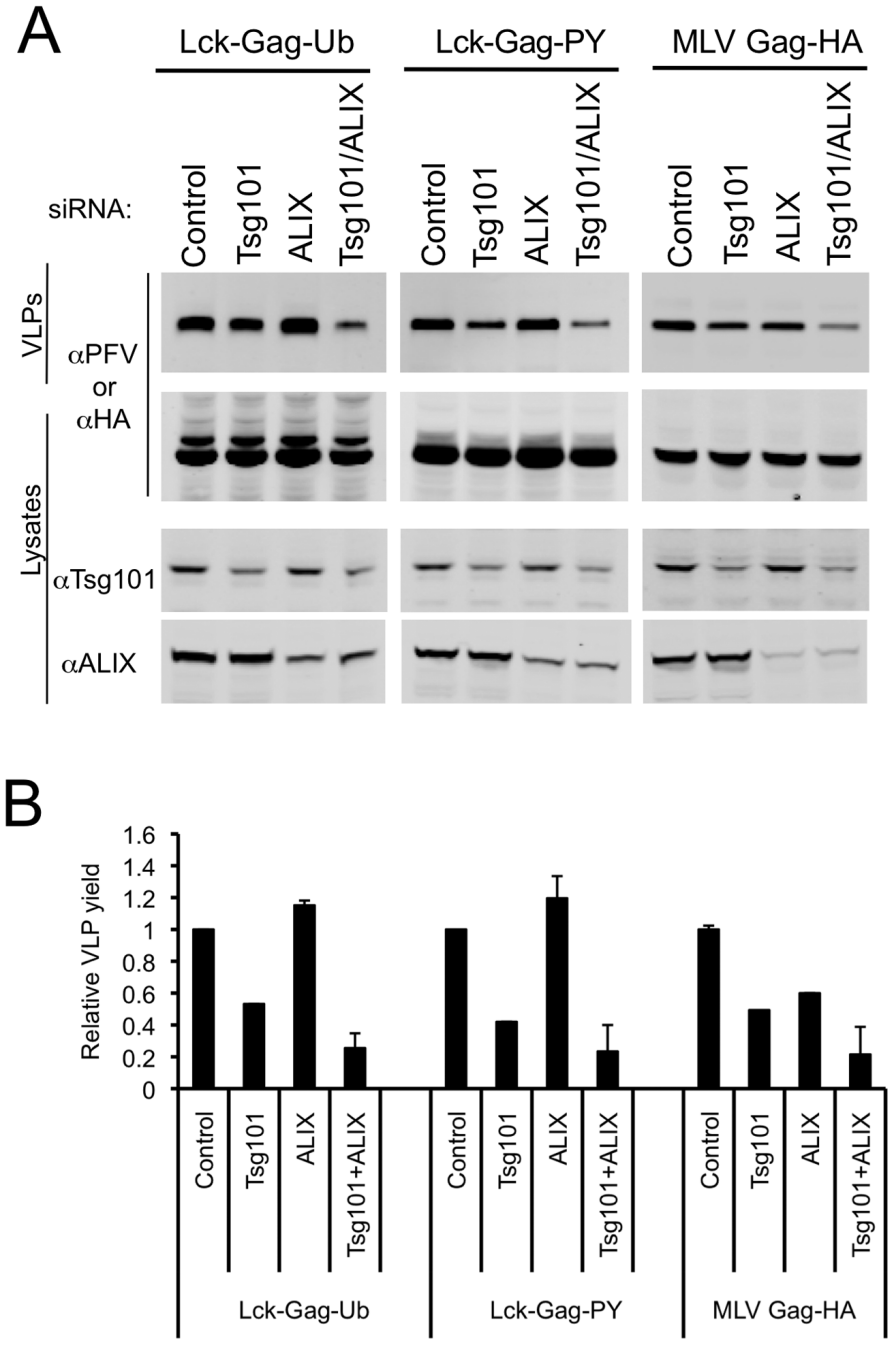
Effect of simultaneous ESCRT-I and ALIX disruption on ubiquitin-dependent and PPxY-dependent particle release. (A) Quantitative Western blot analysis of VLP production from 293T cells transfected with plasmids expressing Lck-Gag-Ub, Lck-Gag-PY or MLV Gag-HA and siRNAs targeting Tsg101, ALIX or both. Corresponding cell lysates were also probed with antibodies to PFV, HA, Tsg101 and/or ALIX, as appropriate, and as indicated. (B) Quantitation of VLP release following knockdown of Tsg101, ALIX or both. Values are plotted as the mean+SD of two or three independent experiments and represent the levels of particles released relative to those released from cells transfected with control luciferase siRNAs, which was assigned a value of 1.

Because ESCRT-I and ALIX perturbation both affected ubiquitin and PPxY-dependent budding, we sought to determine whether their simultaneous depletion would exhibit a stronger inhibitory effect. Unfortunately, cotransfection of the two pools of siRNAs (or each pool together with normalizing control RNA duplexes,) rendered each somewhat less effective, perhaps due to dilution of the active siRNAs (Fig. 8A). Specifically, Tsg101 protein levels were reduced to 42±2% and 50±2% of endogenous levels, while ALIX protein levels were reduced to 30±3% and 27±2% of endogenous levels, when the Tsg101 or ALIX targeted siRNAs were cotransfected together or with normalizing control siRNAs, respectively (Fig. 8A). Thus, under these conditions, siRNAs targeting ALIX did not inhibit Lck-Gag-Ub or Lck-Gag-PY particle release (Fig. 8A,B). Nevertheless, simultaneous (albeit partial) depletion of Tsg101 and ALIX had a significantly stronger inhibitory effect on Lck-Gag-Ub, Lck-Gag-PY and MLV Gag budding (Fig. 8A, B) than did the more effective individual depletion of either Tsg101 or ALIX alone (Fig. 7C, D), suggesting that they both proteins contribute to optimal PPxY and ubiquitin-dependent budding.

## Discussion

The precise role of HECT ubiquitin ligases in promoting PPxY-dependent virion release has, heretofore, been somewhat unclear. Our previous studies suggest that their ubiquitin ligase activity is critical for their ability to stimulate budding [17], [29], but the functionally relevant substrate for ubiquitination has been difficult to define. Additionally, there is some evidence suggesting that HECT ubiquitin ligases may also function as adaptors for bridging factors that recruit ESCRT proteins to assembling virions [17], [32], [51].

We compared the activities of HECT domains from various Nedd4-like family HECT ubiquitin ligases by fusing them to the C2 and WW domains of WWP1. While this strategy does not illuminate which ubiquitin ligases are responsible for viral budding in the natural context, it does allow an assessment of HECT domain function in a uniform background. We found that HECT domains varied significantly in their ability to stimulate PPxY-dependent particle release in this context. This variability was evident when there were no ubiquitin acceptors in the Gag protein and correlated better with the ability of the HECT domains to drive autoubiquitination than with their ability to ubiquitinate a modified Gag substrate that contained lysines proximal to a PPxY motif. The correlation between autoubiquitination and budding was imperfect, however, and it is possible that variation among the HECT domains in their ability to catalyze different lengths and types of ubiquitin chains (e.g. K48 versus K63-linked chains), or their ability to ubiquitinate other bystander proteins, could influence their ability to stimulate viral budding. In this regard it was notable that there was no correlation between the ability of the HECT domains to undergo autoubiquitination versus their ability to catalyze ubiquitin ligation to Lck-Gag-PY-3K. It was nonetheless true that the ability of the HECT domains to stimulate budding was, in every case, absolutely dependent on their ability to catalyze the ligation of ubiquitin to a substrate. This suggests that the proposed role of HECT domains as adaptors that bind directly to downstream factors is of secondary importance in stimulating budding, or that this adaptor function requires catalytic activity. This latter scenario could, conceivably, be operative as a result of HECT autoubiquitination.

**Figure 9:**
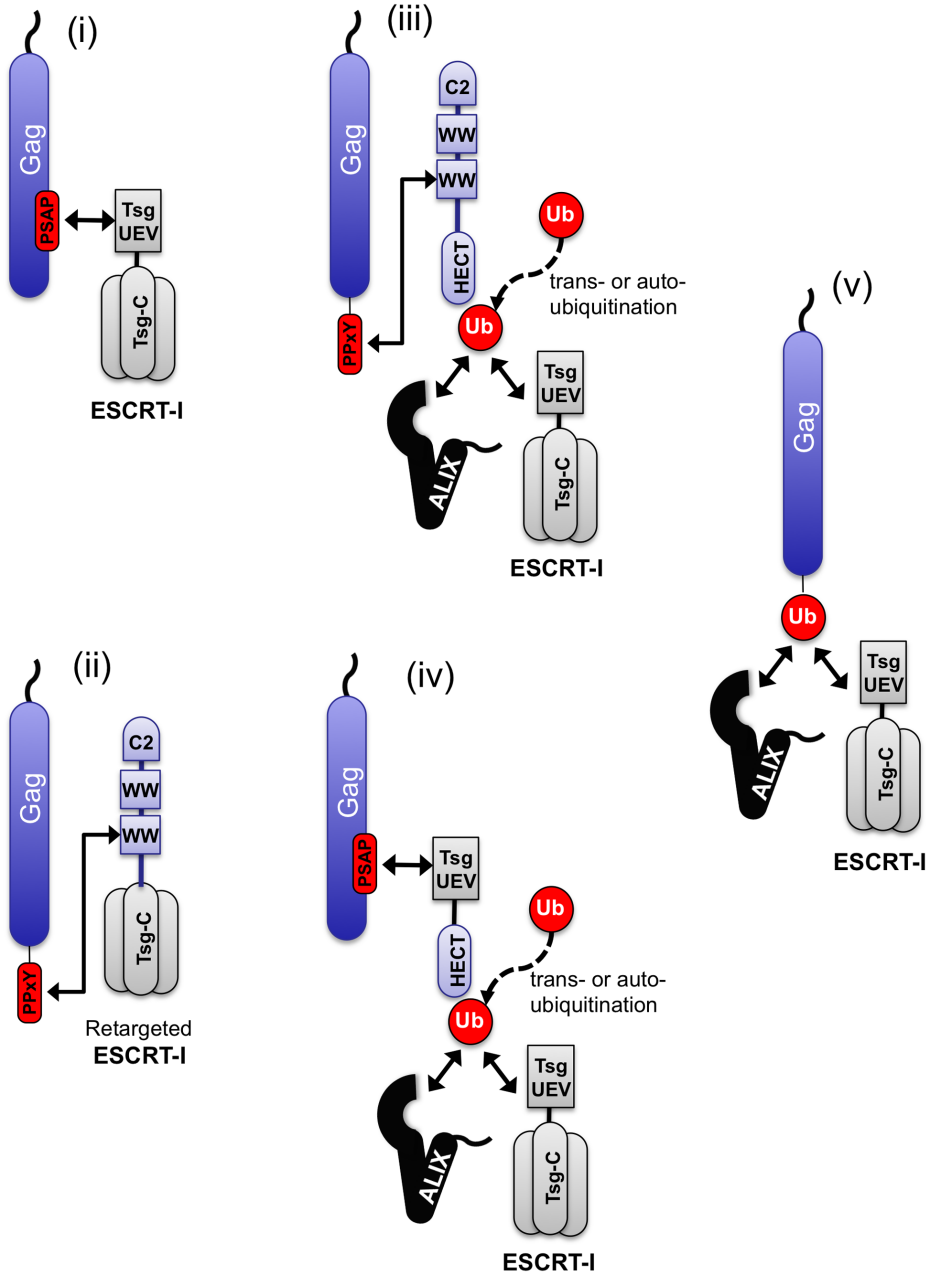
Diversity of pathways that can be used to engage the ESCRT machinery. The ESCRT pathway can be engaged by Gag using a variety of natural and artificial mechanisms (i)–(v) that include ubiquitin ligation to trans-acting proteins, (perhaps including the ubiquitin ligase itself) or fusion to Gag. These studies suggest that ubiquitin functions like a transferable L-domain, recruiting class E VPS factors such as ESCRT-I and ALIX, independently of the identity if the protein to which it is attached.

These studies underscore the remarkable flexibility in the ways that the ESCRT pathway can be engaged to achieve viral budding (Fig. 9) Using a single viral Gag protein as a model, particle budding could be achieved by: (i) conventional direct recruitment of the ESCRT pathway via PTAP binding to Tsg101, (ii) direct recruitment of the ESCRT pathway via PPxY binding to a hybrid cofactor consisting of the C2/WW domains of WWP-1 linked to the C-terminal domain of Tsg101, (iii) recruitment of a HECT ubiquitin ligase via a PPxY motif, (iv) recruitment of an isolated HECT domain to a PTAP motif using a hybrid L-domain cofactor consisting of the UEV domain of Tsg101 linked to a HECT domain or (v) direct fusion of ubiquitin to Gag. These results suggest that the cellular factors (in this case Tsg101, ubiquitin ligases and ubiquitin) that are either directly recruited or deposited at the site of viral particle budding behave as modular entities, with domains that are necessary and sufficient for their own recruitment, and distinct domains that are necessary and sufficient for the subsequent recruitment of downstream effectors of particle release (Fig. 9).

When HECT domains were used to promote budding, the requirement for catalytic activity was absolute, irrespective of how they were recruited to Gag and, importantly, in the absence of ubiquitin acceptors on the viral Gag protein. This finding suggests that ligation of ubiquitin to trans-acting factors, perhaps including the HECT domain itself (i.e. autoubiquitination), rather than Gag is important for viral budding. It is superficially paradoxical, therefore, that ubiquitin could promote budding of the very same Gag protein even when ubiquitin was not ligated to a trans-acting factor, but rather was directly fused to Gag. These findings suggest that the identity of the protein(s) to which ubiquitin is attached is not of critical importance, and ubiquitination substrates can, in principle, include Gag, the ubiquitin ligase itself, or other trans-acting proteins. The mere presence of ubiquitin at the site of particle assembly appears sufficient to engage the ESCRT pathway and stimulate budding.

The intrinsic manipulability of L-domains, the proteins that bind to them (specifically ESCRT-I and HECT ubiquitin ligases) and the apparent lack of importance of the identity of ubiquitination substrate suggests that each serve simply as recruitment factors to engage the downstream machinery that mediates membrane fission and particle release. Since ubiquitin binds to the very same factors (ESCRT-I and ALIX) that are bound by PT/SAP and YPXL type L-domains, and depends on them to stimulate budding, then ubiquitin itself can be conceptually viewed, in the context of viral budding, as a transferable L-domain that acts in a position-independent manner. In essence, this notion is a simple extension of the concept originally demonstrated by Parent et al, who showed that conventional L-domains function in a position independent, transferable manner [56].

A finding that is consistent with the aforementioned arguments, is that budding that was dependent either on a PPxY motif or a ubiquitin fused directly to Gag exhibited similar dependence on particular components of the ESCRT pathway. Notably, perturbation of individual segments of the pathway (ESCRT-I and ALIX) caused partial inhibition of ubiquitin-dependent Lck-Gag-PY, Lck-Gag-Ub and MLV Gag particle release. Previous work has shown that Mason-Pfizer monkey virus particle release, which is dependent on a PPxY motif, is quite strongly inhibited by depletion of Tsg101 [31] and that budding of a EIAV Gag-ubiquitin fusion protein is modestly inhibited by Tsg101 or ALIX depletion [48]. We found that simultaneous perturbation of ESCRT-I and ALIX resulted a stronger suppression of Lck-Gag-PY, Lck-Gag-Ub and MLV Gag particle release than did depletion of either protein alone, suggesting that both ESCRT-I and ALIX can contribute to optimal PPxY- and ubiquitin-dependent budding (Fig. 9). Indeed, the class E VPS pathway includes multiple ubiquitin-interacting factors, each of which could, in principle, provide parallel mechanisms for engaging the ESCRT machinery. While ESCRT-I and ALIX appeared to be most important for PPxY- and ubiquitin-dependent budding, these experiments do not exclude a contributory role for other ubiquitin binding complexes in the ESCRT pathway. A similar notion was recently demonstrated in yeast, where simultaneous disruption of ubiquitin binding by ESCRT-I, -II and Bro1 (the yeast homologue of ALIX) was necessary to block the sorting of ubiquitinated cargo to the lysosome [57]. Thus, ubiquitin has several potential entry points into the ESCRT pathway, and it appears that multiple interactions must be simultaneously inhibited in order to profoundly inhibit ubiquitin- or HECT ligase-dependent budding.

Since ubiquitin-binding class E VPS factors generally have a low affinity for individual ubiquitin molecules (Table 1), the efficiency with which they are recruited to, and retained at, sites of particle assembly is likely related to the number of ubiquitin molecules that are locally present. Indeed, in the context of direct ubiquitin fusion to Lck-Gag, particle release efficiency increased as the proportion of Gag molecules that carried a ubiquitin was increased, and directly fused ubiquitin could effectively bypass the need for a conventional L-domain only when a large fraction (>50%) of the Gag molecules were fused to ubiquitin. This approximates to ∼1000 to 2500 ubiquitin molecules per assembling virion.

Previous studies have shown that direct ubiquitin fusion to RSV or EIAV Gag can alleviate a late budding defect imposed by proteasome inhibitors or functionally replace a YPDL L-domain [44], [48]. However, this study is the first to demonstrate that ubiquitin can act synergistically with a PTAP motif, resulting in dramatically enhanced particle release when both are present. Moreover, the ability of fused ubiquitin to stimulate budding became evident at significantly lower Gag-ubiquitin abundance (5% to 25% of total Gag) when a PTAP motif was also present in Gag. Since ubiquitin could serve as an additional docking site for Tsg101, it might synergize with PTAP motifs by increasing the overall affinity of the assembling Gag lattice for individual ESCRT-I complexes. In fact, this property was predicted by previous binding studies involving Tsg101 UEV domain, PTAP containing peptides and ubiquitin [7]. Ubiquitin might also synergize with PTAP motifs by providing binding sites for distinct class E VPS factors (e.g. ALIX), thereby optimally utilizing all the available components of the ESCRT machinery. Consistent with these ideas, PTAP and PPxY L-domains behaved synergistically in driving particle release, as did PTAP and Gag-fused ubiquitin. However, a PPxY motif and Gag-fused ubiquitin behaved redundantly, consistent with the notion that that they ultimately function through the same mechanism.

## Materials and Methods

### Plasmid construction

pCAGGS-based expression plasmids encoding Lck-Gag(PSAP), Lck-Gag(L-), Lck-Gag-PY, and Lck-Gag-PY-3K plasmids have been described previously [29]. The Lck-Gag(ASAP) plasmid was derived from Lck-Gag(PSAP) by PCR-based site-directed mutagenesis. The Lck-Gag(PSAP)-PY plasmid was generated by replacement of a StuI/XhoI fragment from the Lck-Gag(PSAP) plasmid with the corresponding fragment from the Lck-Gag-PY plasmid. cDNAs expressing Lck-Gag-Ub (ubiquitinΔGG) fusion proteins were generated by overlap-extension PCR, using pCAGGs-Lck-Gag(PSAP), Lck-Gag(L-), and Lck-Gag(PSAP)-PY as templates for the N-terminal portions and pHA-ubiquitin as the template for the C-terminal portion. The K48R, K63R, F4A, L8A, I44A, and QE62,64AA point mutations were introduced into the Lck-Gag-Ub construct by PCR-based mutagenesis. Each cDNA was cloned into pCAGGs for expression in mammalian cells.

DNAs encoding the HECT domains from WWP1 (residues 543–922), WWP2 (491–870), Nedd4 (520–902), Nedd4L (593–975), Itch (483–862), Smurf1 (374–757), and Rsp5 (431–809) were amplified from plasmids encoding the full-length HECT ligases [17], [58]. The Bul2 HECT domain (encoding residues 1189–1572) was PCR amplified from 293T cell cDNA. The catalytically inactive WWP1 HECT domain (C890S) was amplified from a previously described full-length mutant WWP1 ligase [17]. Catalytic point mutants of the remaining HECT domains were made by PCR-based mutagenesis. Chimeric ubiquitin ligases, comprising the C2 and WW domains (residues 1–542) of WWP1 and each of the HECT domains described above were generated by overlap PCR. Likewise, plasmids expressing Tsg-WWP1, Tsg-Nedd4L and Tsg-Itch (residues 1–157 of Tsg101 fused to HECT domains of WWP1, Nedd4L, or Itch) as well as WWP1-Tsg-C (residues 1–542 of WWP1 fused to residues 157–390 of Tsg101) were constructed by overlap-extension PCR. All cDNAs encoding chimeric proteins were inserted into pCR3.1/YFP, to express proteins fused to the C-terminus of YFP, for in mammalian cells. The class E VPS factor yeast two-hybrid library and plasmids expressing Vps4 E228Q, Tsg101, Hrs, ALIX, UBPY, and Eap45 fluorescent fusion proteins in mammalian cells have been described previously [8], [12], [54]. Yeast two-hybrid plasmids encoding wild type and I44A mutant ubiquitin were constructed by PCR amplification of ubiquitinΔGG from the pHA-ubiquitin plasmid using 5′ and 3′ primers appended with EcoRI restriction sites and cloning into the pGBKT7 (Clontech) and pVP16 vectors [8].

### Virus release assays

For Gag particle release assays, 5×10^5^ 293T cells in six-well plates were transfected using polyethylenimine (Polysciences) with 1 µg of pCAGGs/Gag-derived plasmids, alone or with 1 µg of pCR3.1/YFP, pCR3.1/YFP-WWP1/HECT, pCR3.1/YFP-Tsg-HECT, or pCR3.1/YFP-C2-WW-Tsg-C plasmids, or the indicated amounts of pCR3.1/YFP-Vps4 E228Q plasmid. For EIAV and MLV VLP release assays, 293T cells were transfected in the same format with 500ng of, pCR3.1/EIAVGag or pCR3.1/MLVGag-HA plasmids. VLPs were pelleted by ultracentrifugation of 2 ml of 0.22-µm-filtered culture supernatants, collected 48 hours after transfection, over a 2ml 20% sucrose cushion for 90 min at 115,000×g. VLP and cell lysates were analyzed by Western blotting.

### Ubiquitination assay

293T cells (5×10^5^) in six-well plates were cotransfected with 1 µg of pCAGGs/Lck-Gag-PY-3K, 500 ng of pHA-ubiquitin, and 1 µg of the indicated chimeric pCR3.1-WWP1-HECT ligase. At 36h after transfection, cells were thoroughly lysed at room temperature in detergent-rich RIPA buffer (50mM Tris pH 7.4, 150mM NaCl, 1mM EDTA, 1.0% glycerol, 0.5% SDS, supplemented with protease inhibitor tablets (Roche) and 5mM N-ethylmaleimide to inhibit deubiquitination) and cleared of cellular debris by microcentrifugation. The lysates were then diluted 5-fold in the same buffer containing NP-40 rather than SDS, to adjust the concentration of SDS to 0.1% and NP-40 to 1.0%, and split into two fractions. From one fraction, Gag proteins were immunoprecipitated with αPFV serum and protein G-Sepharose beads. From the other fraction, YFP-HECT ligase proteins were immunoprecipitated with αGFP monoclonal antibody and protein G-Sepharose beads. Immunoprecipitates and unfractionated cell lysates were analyzed by Western blotting.

### siRNA transfections

293T cells (3×10^5^) in six-well plates were transfected with siGENOME siRNAs targeting Luciferase, Tsg101, Hrs, Alix, UBPY, or Eap45 (Dharmacon) using Lipofectamine 2000 (Invitrogen). After 24h, cells were transfected with the same siRNAs and the indicated Gag expression plasmids. VLP and cell lysates were prepared 48 h after the second transfection. To assess knockdown efficiency, 293T cells were transfected once with YFP-Tsg101, -Hrs, -ALIX, -UBPY, or -Eap45 expression plasmids and corresponding siRNAs. GFP expression in cell lysates harvested 48 h after transfection was assayed by quantitative Western blotting.

### Western blot analyses

Virion and cell lysates and immunoprecipitates were separated on polyacrylamide gels, transferred to nitrocellulose membranes, and probed with various antibodies: anti-PFV human serum, anti-HIV-1 p24CA (183-H12-5C), anti-EIAV equine serum (VMRD, Inc.), anti-GFP (Roche), and anti-HA (HA.11, Covance) anti-Tsg101 (4A10, Abcam, Cambridge, MA) or anti-ALIX rabbit serum (a gift from Wes Sundquist). Subsequently, the blots were probed with species-specific peroxidase-conjugated secondary antibodies and chemiluminescent substrate reagents. Alternatively, for quantitative Western blotting, membranes were probed with species-specific antibodies conjugated to IRDye800CW, and fluorescent signals were detected and quantified using a LICOR Odyssey scanner.

### Yeast two-hybrid analyses

Yeast cells (Y190) were transformed with the pGBKT7- and pVP16-derived plasmids described above. Transformants were selected and protein-protein interactions were assayed by β-galactosidase reporter activity as previously described [8].
